# THE PARADOX OF CONNECTION: MUTUAL FOLLOWING’S IMPACT ON LONELINESS AMONG OLDER ADULTS IN ONLINE NETWORKS

**DOI:** 10.1093/geroni/igae098.0673

**Published:** 2024-12-31

**Authors:** Nahyun Kim, Keiko Katagiri

**Affiliations:** Kobe University, Kobe, Hyogo, Japan; Kobe University, Kobe, Hyogo, Japan

## Abstract

Online communities serve as a resource for many older adults to create new connections, such as friendships and acquaintanceships that can help alleviate loneliness. Despite the many advantages, there is a lack of research on the network size in online communities tailored for older adults and its effects on loneliness reduction. This study examines mutual following on online platforms as an indicator of new connections, focusing on its relationship with the perceived formation of new connections and their subsequent effect on loneliness reduction among community members. In February 2023, we conducted an online survey within an online community for older adults in Japan, involving participants aged 60–79 (n=819). The platform provided features such as photo sharing, group participation, and user following. Data collected included group participation, mutual following, perceived establishment of new online connections, and loneliness. A path analysis revealed that a greater number of mutual followers was positively related to the perception of establishing new online connections, leading to a reduction in loneliness. However, we also found a direct positive association between mutual following and loneliness, indicating a paradoxical finding: the mere increase in mutual followers, devoid of meaningful engagement, may exacerbate loneliness. This suggests that the effectiveness of online connections in reducing loneliness is contingent upon the quality of relationships formed. These findings highlight the importance of fostering quality interactions within online communities, to enhance older adults’ well-being in digital environments.

